# Supplementary figures and images for: Serum Uric Acid Is Associated with Insulin Resistance in Non-Diabetic Subjects
Source: J Clin Med. 2025 Apr 11;14(8):2621. doi: 10.3390/jcm14082621 (PMC12028212; doi:10.3390/jcm14082621)

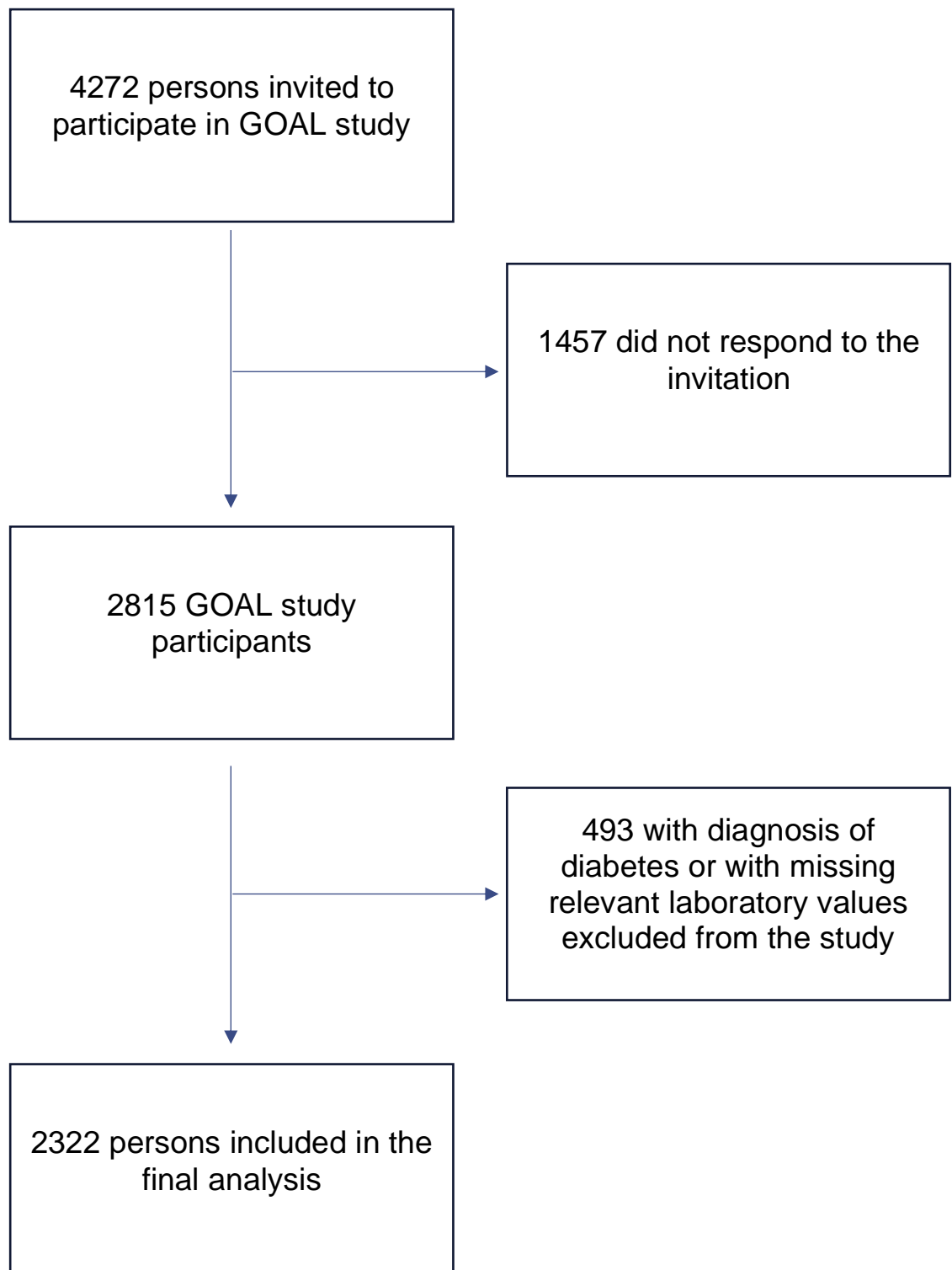

Figure S1. Flowchart detailing the selection of study subjects.

Supplement: Supplementary file 1 [file jcm-14-02621-s001.zip › jcm-3506065-supplementary.pdf]
